# Supplementary material for: Boutiques: a flexible framework to integrate command-line applications in computing platforms
Source: Gigascience. 2018 Mar 23;7(5):giy016. doi: 10.1093/gigascience/giy016 (PMC6007562; doi:10.1093/gigascience/giy016)
Supplement: Response_to_Reviewer_Comments_Original_Submission.pdf [file giy016_response_to_reviewer_comments_original_submission.pdf]

Author's Response To Reviewer Comments

See detailed answers in letter attached.
