# Supplementary material for: Boutiques: a flexible framework to integrate command-line applications in computing platforms
Source: Gigascience. 2018 Mar 23;7(5):giy016. doi: 10.1093/gigascience/giy016 (PMC6007562; doi:10.1093/gigascience/giy016)
Supplement: Response_to_Reviewer_Comments_Revision_1.pdf [file giy016_response_to_reviewer_comments_revision_1.pdf]

Author's Response To Reviewer Comments

Dear Editors,

In response of your email on February 19, we resubmit this paper after having implemented the following requested modifications:

- Added a section on "Availability of supporting source code and requirements" that lists the project name, RRID, home page, operating system, programming language, other requirements and license.

- Added a section on "Availability of supporting data" that mentions that there is a version of the code on Zenodo and cites the exact version used in the paper.

- Registered Boutiques in the SciCrunch.org database and received an RRID.

We thank you again for considering our paper for publication in GigaScience.

Sincerely,

Tristan Glatard,
on behalf of the authors
