# Supplementary material for: Boutiques: a flexible framework to integrate command-line applications in computing platforms
Source: Gigascience. 2018 Mar 23;7(5):giy016. doi: 10.1093/gigascience/giy016 (PMC6007562; doi:10.1093/gigascience/giy016)
Supplement: Reviewer_2_Report_(Original_Submission) -- Jakub Kaczmarzyk — 04 Dec 2017 Reviewed [file giy016_reviewer_2_report_(original_submission).pdf]

Reviewer Report

Title: Boutiques: a flexible framework to integrate command-line applications in computing platforms

Version: Original Submission **Date:** 04 Dec 2017

Reviewer name: Jakub Kaczmarzyk

Reviewer Comments to Author:

Boutiques is a specification that abstracts command-line programs, their arguments, and their expected outputs. The goal of the specification is to simplify application integration, reduce code redundancy, and support reproducible science. Following the Boutiques specification, a command-line program is documented in a descriptor file, which follows the JSON specification. A descriptor file specifies the arguments and expected outputs of a program. The file includes a template of the command-line instruction, the location of a software container that includes the program, information about each parameter (e.g., what type of argument it expects, if any), and relationships within groups of related parameters (e.g., parameters in a group might be mutually exclusive). Potential arguments to a program may be validated against the information in that program's descriptor. Early validation is a key feature of the Boutiques specification, because it identifies issues to the user prior to the program's runtime. The command-line template is also advantageous because it allows for shell functions to be used, like piping and input/output redirection. As a side effect of the level of detail required by the Boutiques specification, the descriptor file also serves as precise usage documentation of a program.

Moreover, the paper describes the Boutiques core tools, which I understood to be a Python implementation of the Boutiques specification. These core tools include `bosh`, a command-line program which enables users to validate Boutiques descriptors and invocation files, easily run containerized programs, convert BIDS apps to Boutiques descriptors, and publish their Boutiques descriptors to the NeuroLinks website. `bosh` can run containers in Docker, Singularity, or rootfs format. This feature satisfies the needs of both local execution and execution on high performance computing clusters. The inclusion of containers in the Boutiques specification lessens the burden on the user because the user does not have to install the software on the host machine.

I would recommend a minor revision of this paper. The descriptor schema and core tools were described and reasoned well, but the overall goal of Boutiques could be clarified earlier in the paper. I found the last sentence of the section titled "Other frameworks" to be a concise and informative summary of the goal of Boutiques and the advantages of Boutiques over other, similar frameworks. The authors might consider adding a similar sentence to the introduction of the paper.

The work could also benefit from minor clarification of the descriptor specification and minor updates to the core tools. Overall, I found the descriptor schema well-documented, intuitive, modular, and relatively complete (keeping in mind the ability to specify custom parameters). I also found the various listings in the paper and the online documentation helpful. I installed Boutiques version 0.5.3 (Python

3.6.3 on OS X 10.11.6) to test the minimal Boutiques descriptor in Listing 5. This descriptor passed the validation step, but when attempting to conduct a simulation run of the descriptor given an input JSON file with the contents `{"param": 5}`, a `KeyError` was produced, indicating that the key "container-image" was not present in the descriptor. This led me to wonder whether "container-image" is a required field in the descriptor schema and whether `bosh` can execute on bare metal.

When trying to run the example in https://github.com/boutiques/boutiques/tree/https://github.com/boutiques/boutiques/tree/5678d6f84bc1863741c6b8c595f037f622936c5c/tools/python/boutiques/schema/examples/example1/tools/python/boutiques/schema/examples/example1, validation of the invocation and descriptor functioned as described. For instance, validation failed when `enum_input` and `num_input`, mutually exclusive parameters, were both specified. Validation did not fail, however, when attempting to run a 'simulation' or 'launch' with the same invalid inputs. The authors should consider including the validation step when running the program with `bosh exec`.

Furthermore, the feature of `bosh` to upload descriptors to a common repository (i.e., NeuroLinks) is an important one. While links to descriptors are certainly useful, I would like to suggest a common repository of the descriptor files themselves. Such a repository would reduce redundancy in coding effort because contributors could see which programs already have descriptors. Upstream software maintainers could maintain their Boutiques descriptors in such a repository. The burden on new users might also be reduced because they would have access to all Boutiques descriptors in a single location. If a common repository like this were in place, `bosh` might be extended to accept descriptors in a more abstract form. For example, instead of specifying a path to an existing descriptor file, a user could specify "fsl.bet.v1", and `bosh` could pull the relevant descriptor from the common repository. A similar effect might be achieved using the existing JSON file that lists the tools on the NeuroLinks website.

In conclusion, Boutiques is a framework that flexibly specifies command-line programs and improves reproducibility through the use of software containers. Other frameworks can leverage Boutiques to create self-contained workflows that only depend on a container implementation like Docker or Singularity.

Level of Interest

Please indicate how interesting you found the manuscript: An article of importance in its field

Quality of Written English

Please indicate the quality of language in the manuscript: Acceptable

Declaration of Competing Interests

Please complete a declaration of competing interests, considering the following questions:

- Have you in the past five years received reimbursements, fees, funding, or salary from an organisation that may in any way gain or lose financially from the publication of this manuscript, either now or in the future?
- Do you hold any stocks or shares in an organisation that may in any way gain or lose financially from the publication of this manuscript, either now or in the future?
- Do you hold or are you currently applying for any patents relating to the content of the manuscript?
- Have you received reimbursements, fees, funding, or salary from an organization that holds or has applied for patents relating to the content of the manuscript?
- Do you have any other financial competing interests?
- Do you have any non-financial competing interests in relation to this paper?

If you can answer no to all of the above, write 'I declare that I have no competing interests' below. If your reply is yes to any, please give details below.

I declare that I have no competing interests.

I agree to the open peer review policy of the journal. I understand that my name will be included on my report to the authors and, if the manuscript is accepted for publication, my named report including any attachments I upload will be posted on the website along with the authors' responses. I agree for my report to be made available under an Open Access Creative Commons CC-BY license (http://creativecommons.org/licenses/by/4.0/). I understand that any comments which I do not wish to be included in my named report can be included as confidential comments to the editors, which will not be published.

I agree to the open peer review policy of the journal

To further support our reviewers, we have joined with Publons, where you can gain additional credit to further highlight your hard work (see: https://publons.com/journal/530/gigascience). On publication of this paper, your review will be automatically added to Publons, you can then choose whether or not to claim your Publons credit. I understand this statement.

Yes
